# Analysis of Distortion Based on 2D MEMS Micromirror Scanning Projection System

**DOI:** 10.3390/mi12070818

**Published:** 2021-07-13

**Authors:** Xichen Wang, Yingke Xie, Hengheng Liang, Nianbing Zhong

**Affiliations:** 1 Liangjiang International College, Chongqing University of Technology, Chongqing 401135, China; wangxichen@2019.cqut.edu.cn; 2 Research Center of LIDARs and Intelligent Optical Nodes, Chongqing University of Technology, Chongqing 401135, China; lianghengheng@2019.cqut.edu.cn; 3 College of Science, Chongqing University of Technology, Chongqing 401135, China

**Keywords:** 2D MEMS micromirror, distortion, scanning projection

## Abstract

To analyze the distortion problem of two-dimensional micro-electromechanical system (MEMS) micromirror in-plane scanning, this paper makes a full theoretical analysis of the distortion causes from many aspects. Firstly, the mathematical relations among the deflection angle, laser incidence angle, and plane scanning distance of the micromirror are constructed, and the types of projection distortion of the micromirror scanning are discussed. Then the simulation results of reflection angle distribution and point cloud distribution are verified by MATLAB software under different working conditions. Finally, a two-dimensional MEMS micromirror scanning projection system is built. The predetermined waveform can be scanned and projected successfully. The distortion theory is proved to be correct by analyzing the distortion of the projection images, which lays a foundation for practical engineering application.

## 1. Introduction

The MEMS scanning micromirror was first proposed by Peterson [1] in the 1980s. Since the start of the 21st century, scanning technology based on MEMS micromirror has made great progress. It has been extremely widely used, especially in laser radar [2], optical display [3,4], and micromirror imaging [5,6]. MEMS micromirror has the advantages of a light weight, small size, and low cost. Compared with traditional projection equipment, it has great advantages in applying laser projection display [7]. A laser light source is needed to scan the reflected beam through the MEMS 2D micromirror to form a 2D plane projected onto the imaging plane to obtain the scan pattern [8]. The introduction of the first micromirror device product from TI in 1998 [9] made the MEMS scanning micromirror projection display a great success in the market. Therefore, the planar scanning technology for MEMS micromirrors has gradually become a hot topic at home and abroad.

However, in the application of 2D MEMS micromirrors for large-scale scanning projection, due to the extremely limited mirror size and deflection angle of MEMS micromirrors [10], higher demands are required for the divergence angle of the scanning laser source, the size of the emitted spot, and the incident angle, resulting in a certain degree of distortion of the projected pattern compared with the preset waveform. In demanding projection effects, this distortion will affect the effectiveness of the subsequent processing of graphics or data acquisition systems. Now, the MEMS micromirror scanning technology is becoming more and more widely used. Therefore, it is more and more important to study the various distortions that occur in the micromirror scanning process. Sidler analyzed the scanning distortion of biaxial tilted single-mirror scanners and concluded that the incident angle was the most important factor affecting the scanning optical path distortion, which laid a foundation for the study of plane projection scanning distortion [11]. However, the optical scanning model has not been thoroughly analyzed, and the types of distortion have not been identified. To investigate the causes and laws of scanning projection distortion, this paper discusses the detailed theoretical analysis of scanning distortion and recognizes the types of scanning distortion for the optical scanning principle of MEMS micromirror, building a 2D MEMS micromirror scanning projection platform to show the distortion effect. It provides theoretical support for subsequent studies on the distortion of MEMS micromirror planar scanning.

## 2. Principle of 2D MEMS Micromirror Scanning Projection

### 2.1. Working Principle of Reflective Micromirror

**Figure 1:**
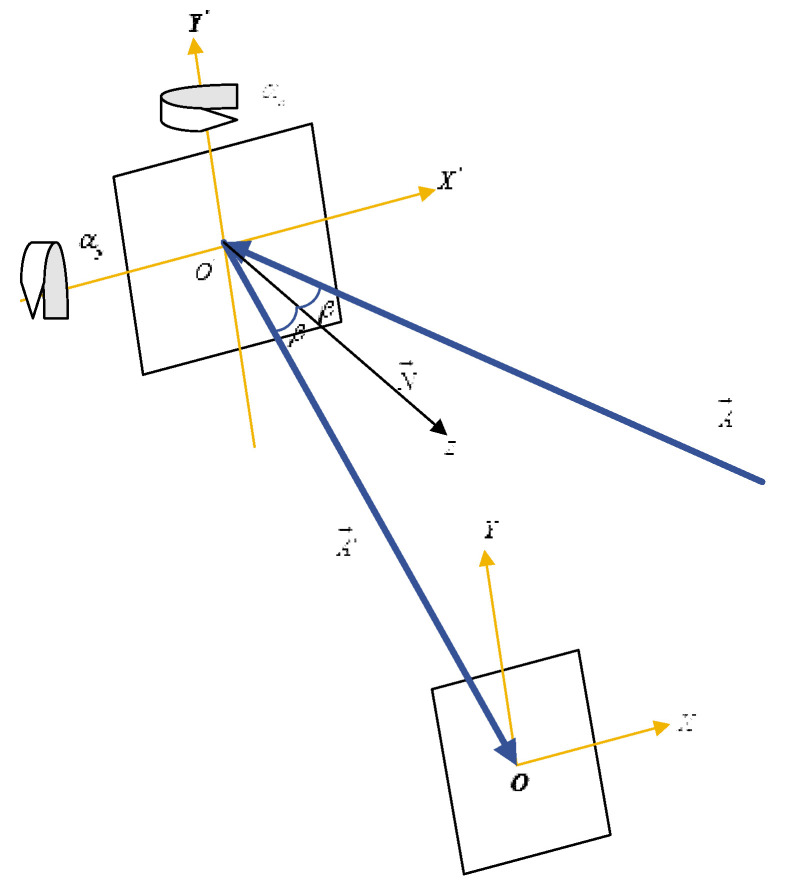
Cartesian coordinate system based on MEMS micromirror scanning.

To investigate the relationship between the deflection of the 2D MEMS micromirror and the waveform of planar scanning, Figure 1 shows the established spatial right-angle coordinate system. In the initial state when the 2D MEMS micromirror is not vibrating, the MEMS mirror is coincident with the X’O’Y’ plane and $\vec{N}$ is the normal unit vector of the X’O’Y’ plane coincident with the $Z^{\prime }$ axis. The incident light $\vec{A}$ is incidentally at an angle of β to the MEMS reflection plane, located in the X’O’Z’ plane. The reflected light $\vec{A^{\prime }}$ is reflected by the MEMS reflection plane and enters the XOZ plane, which is coplanar with the X’O’Z’ plane, and the distance of OO’ is *L*. The normal unit vector is $\vec{N}$ and the outgoing ray is $\vec{A^{\prime }}$. The MEMS reflector surface varies continuously with the vibration of the 2D MEMS micromirror in both horizontal and vertical directions, and the relationship is derived by using the method of matrix optics [12].

The vector form of the reflection law can be written in matrix optics [12] as

(1) $$\vec{A^{\prime }}=\vec{A}-2(\vec{A}\cdot \vec{N})\cdot \vec{N}$$

where $\vec{A}$ is the incident light unit vector, $\vec{N}$ is the normal light unit vector, and $\vec{A^{\prime }}$ is the reflected light unit vector.

The vector form of the law of reflection in Equation (1) is transformed into a matrix expression, and a three-dimensional space rectangular coordinate system *x*,*y*,*z* is established.

(2) $$\begin{array}{l} \vec{A}=A_{x}\vec{i}+A_{y}\vec{j}+A_{z}\vec{k}\\ \vec{A^{\prime }}=A_{x}^{\prime }\vec{i}+A_{y}^{\prime }\vec{j}+A_{z}^{\prime }\vec{k}\\ \vec{N}=N_{x}\vec{i}+N_{y}\vec{j}+N_{z}\vec{k} \end{array}$$

Equation (1) can be written as the following relation for the incident ray ***A’***, incident light ***A***, and reflection matrix ***V***:

(3) $$A^{\prime }=VA$$

Substituting Equation (2) into Equation (1),

(4) $$V=\left[\begin{array}{ccc} 1-2N_{x}^{2} & -2N_{y}N_{x} & -2N_{z}N_{x}\\ -2N_{x}N_{y} & 1-2N_{y}{}^{2} & -2N_{z}N_{y}\\ -2N_{x}N_{z} & -2N_{y}N_{z} & 1-2N_{z}{}^{2} \end{array}\right]$$

From Equation (4), it can be found that the expression of ***V*** is only related to the components of the normal unit vector $\vec{N}$ of the reflecting surface in each direction, and the reflection matrix ***V*** is determined by the spatial position of the reflecting surface. When the MEMS micromirror is not vibrating in the initial state, the following relationship can be obtained with XYZ as the main coordinate system:

(5) $$\vec{A}=\left[\begin{array}{l} A_{x}\\ A_{y}\\ A_{z} \end{array}\right]=\left[\begin{array}{c} -\sin2\beta \\ 0\\ -\cos2\beta \end{array}\right]$$

(6) $$\vec{N}=\left[\begin{array}{l} N_{x}\\ N_{y}\\ N_{z} \end{array}\right]=\left[\begin{array}{c} \sin\beta \\ 0\\ \cos\beta \end{array}\right]$$

By substituting Equations (5) and (6) into the reflection matrix and Equation (3), the reflected light unit vector in the initial state is obtained as

(7) $$A^{\prime }=VA=\left[\begin{array}{ccc} \cos2\beta & 0 & -\sin2\beta \\ 0 & 1 & 0\\ -\sin2\beta & 0 & -\cos2\beta \end{array}\right]\left[\begin{array}{c} -\sin2\beta \\ 0\\ -\cos2\beta \end{array}\right]=\left[\begin{array}{l} 0\\ 0\\ 1 \end{array}\right]$$

Equation (7) confirms that the intersection of the outgoing ray $\vec{A^{\prime }}$ and the XOY plane in the initial state is the origin O and the outgoing ray overlaps with the *z*-axis. When the MEMS micromirror starts to vibrate and deflect in two dimensions, the above theory will be used to calculate the coordinates of the intersection of the projection line with the XOY plane and the angle of deflection for the *z*-axis. Assume that the horizontal deflection angle of the MEMS micromirror is $\alpha_{x}$ and the vertical deflection angle is $\alpha_{y}$. It is equivalent to X’O’Y’ a plane in Figure 1, which first rotates counterclockwise $\alpha_{y}$ with the *y*-axis as the rotation axis and then rotates clockwise $\alpha_{x}$ with the *x*-axis as the rotation axis. The new normal unit vector corresponding to the MEMS micromirror reflective surface is

(8) $$\vec{N}=\left[\begin{array}{c} N_{x}\\ N_{y}\\ N_{z} \end{array}\right]=\left[\begin{array}{c} \sin(\beta +\alpha_{x})\cos\alpha_{y}\\ \sin\alpha_{y}\\ \cos(\beta +\alpha_{x})\cos\alpha_{y} \end{array}\right]$$

After calculation and simplification, we can obtain the outgoing ray in the deflected state of the reflecting mirror:

(9) $$A^{\prime }=VA=\left[\begin{array}{c} \cos^{2}\alpha_{y}\sin2\alpha_{x}-\sin^{2}\alpha_{y}\sin2\beta \\ \cos(\beta -\alpha_{x})\sin2\alpha_{y}\\ \cos^{2}\alpha_{y}\cos2\alpha_{x}-\sin^{2}\alpha_{y}\cos2\beta \end{array}\right]$$

Regardless of the deflection of the reflecting mirror, the starting point of the outgoing ray is the point O’(0,0,−*L*), so the linear equation of the outgoing ray is

(10) $$\frac{X}{A_{x}^{\prime }}=\frac{Y}{A_{y}^{\prime }}=\frac{Z+L}{A_{z}^{\prime }}$$

Then the coordinates of the intersection of the incident ray and the XOY plane can be found as

(11) $$\{\begin{array}{c} X=L\frac{\cos^{2}\alpha_{y}\sin2\alpha_{x}-\sin^{2}\alpha_{y}\sin2\beta }{\cos^{2}\alpha_{y}\cos2\alpha_{x}-\sin^{2}\alpha_{y}\cos2\beta }\\ Y=L\frac{\cos(\beta -\alpha_{x})\sin2\alpha_{y}}{\cos^{2}\alpha_{y}\cos2\alpha_{x}-\sin^{2}\alpha_{y}\cos2\beta } \end{array}$$

From Equation (11), when $\alpha_{y}=0$, the MEMS micromirror is deflected only in the horizontal direction, which gives $X=L\tan2\alpha_{x}$, which indicates that the new reflected ray is deflected by an angle of $2\alpha_{x}$ concerning the original reflected ray. That means that the optical reflection angle in the horizontal direction is twice the mechanical deflection angle of the mirror. On the contrary, the relationship is not satisfied in the vertical direction when $\alpha_{x}=0$. Because the condition for this conclusion is that the starting incident light is incident horizontally, it lies on the transverse plane. When $\alpha_{x}=\alpha_{y}=0$ and the vibration amplitude is small, we can think that sinα=0 and cosα=0. Substituting the simplified result into Equation (11), Y/X≈cosβ. The equation indicates that when the mechanical deflection angle is small, the ratio of the vertical scan length of the reflector to the horizontal scan length is approximately the cosine of the angle of incidence of the light, which means that the scanning effect of the reflector is related to the angle of incidence of the starting incident light.

From Equation (11), it can be seen that for different horizontal mechanical deflection angles $\alpha_{x}$ and vertical mechanical deflection angles $\alpha_{y}$ of the MEMS reflector and different initial light incidence angles β, the horizontal deflection angle $\varphi_{x}$ and the vertical deflection angle $\varphi_{y}$ of the outgoing light concerning the initial outgoing light vector are expressed as shown in Equation (12):

(12) $$\{\begin{array}{c} \varphi_{x}=\tan^{-1}\frac{X}{L}=\frac{\cos^{2}\alpha_{y}\sin2\alpha_{x}-\sin^{2}\alpha_{y}\sin2\beta }{\cos^{2}\alpha_{y}\cos2\alpha_{x}-\sin^{2}\alpha_{y}\cos2\beta }\\ \varphi_{Y}=\tan^{-1}\frac{Y}{L}\frac{\cos(\beta -\alpha_{x})\sin2\alpha_{y}}{\cos^{2}\alpha_{y}\cos2\alpha_{x}-\sin^{2}\alpha_{y}\cos2\beta } \end{array}$$

**Figure 2:**
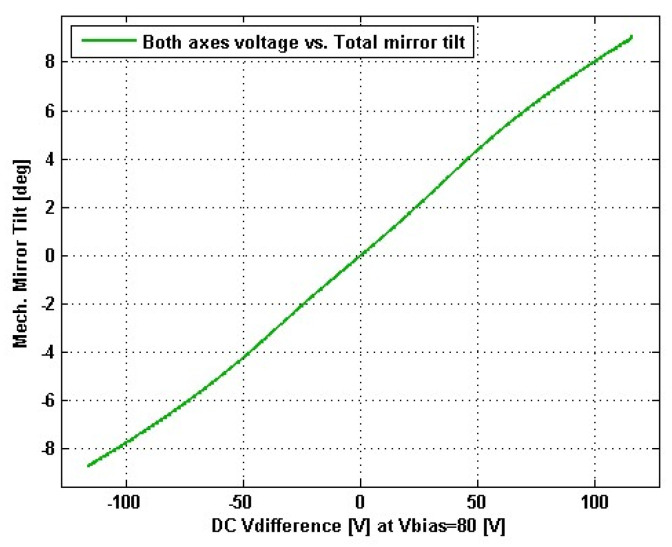
Voltage vs. mech. angle—coupled axes of the MEMS micromirror (Supplemental Materials).

From Equations (11) and (12), we can see that the scanning point position distribution and the angle of the outgoing light in the scanning plane are determined by the micromirror deflection angle, the incident light angle, and the scanning distance together. The micromirror deflection angle varies linearly with voltage, as shown in Figure 2. Thus, the point cloud distribution and the angle of the incident light will occur in a nonlinear distortion situation. The distortion of the scanned image has the same manifestation as the distortion of the camera image [12], where the pillow distortion is due to the distortion of the image showing compression toward the center; the skew distortion is due to the distortion of the image showing compression toward the edges.

### 2.2. Analysis of Scanning Distortion

**Figure 3:**
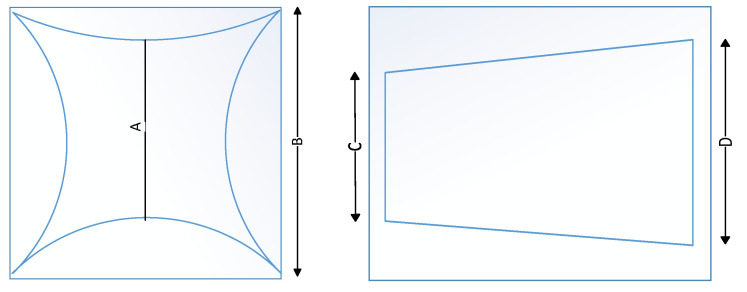
Distortion graphic outline (left: pillow distortion schematic; right: skew distortion schematic).

From the analysis of the 2D MEMS scanning micromirror reflection scanning principle in Section 2.1, it is clear that when applying the MEMS micromirror for planar scanning, the scanning effect is affected by the angle of the incident light and the mechanical deflection angle of the vibrating mirror. From Equation (11), the distribution of scanning points in the scanning plane thus produces a distortion effect; from Equation (12), the deflection angle of the outgoing light also presents a distortion effect. The resulting distortion effect can be divided into two categories, namely pillow distortion and skew distortion. The rough distribution profile is shown in Figure 3.

The pillow distortion rate and the tilt distortion rate can be calculated as follows:

(13) $$R_{pillow\;}=\left(B-A\right)/B$$

(14) $$R_{skew}=\left(D-C\right)/D$$

where *A* is the center length of the pillow distortion graph, *B* is the edge length of the pillow distortion graph, *C* is the narrow edge length of the skew distortion graph, and *D* is the wider edge length of the skew distortion graph.

**Figure 4:**
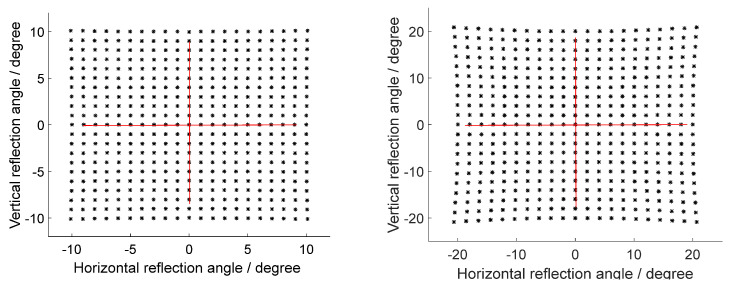
The variation of the deflection angle of the outgoing light with the deflection angle of the MEMS micromirror in both horizontal and vertical directions (the deflection angle of the left figure is ±5°; the deflection angle of the right figure is ±10°).

When the incident light is incident on the reflector surface, the angle of incidence is β=0°, and the angle of deflection of the outgoing light is roughly twice the mechanical angle of the reflector; in addition, the angle of deflection of the outgoing light will exhibit a pillow distortion. As the effect in Figure 4 shows, when the mechanical deflection angle of the MEMS micromirror is ±5°, the pillow distortion rate is about 1.1%. When the mechanical deflection angle of the MEMS mirror is ±10°, the pillow distortion rate is about 4.6%. Moreover, the pillow distortion situation becomes more obvious as the mechanical deflection angle of the mirror increases.

**Figure 5:**
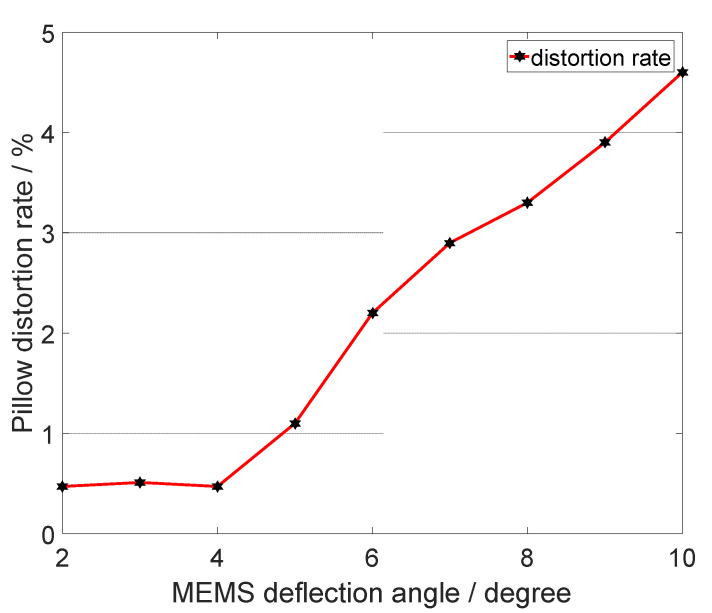
The pillow distortion of reflected light angle varies with the MEMS deflection angle.

At different deflection angles of the 2D MEMS micromirror, the angle of the reflected light increases significantly with the deflection angle of the mirror when the incident angle is not changed. As shown in Figure 5, the pillow distortion of the reflected light increases from 0.47 to 4.6% when the deflection angle of the MEMS micromirror changes from ±2° to ±10°. The distortion will cause a certain range of distortion effects when applying the MEMS micromirror for scanning open and closed-loop feedback and wide range projection.

**Figure 6:**
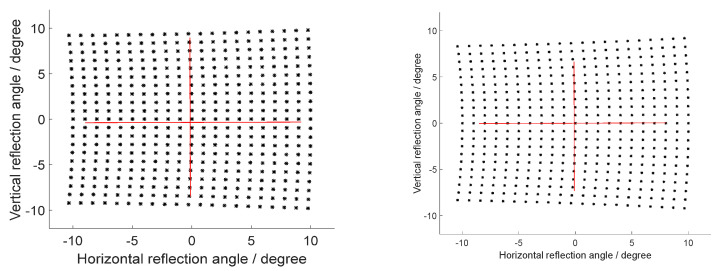
The reflection angle at the different incident angles (the incident angle of the left is 20°; the incident angle of the right is 30°).

At the same mechanical deflection angle of MEMS micromirror, when the incident light is incident to the MEMS reflection plane at different angles, the deflection angle of the reflected light changes with the change of the incident angle. As shown in Figure 6, the deflection angle of the outgoing light produces a skew distortion in addition to a pillow distortion at this moment. The skew distortion is manifested in a dense dot pattern on the left side and a sparse dot pattern on the right side. The change of the incident angle brings about the cause of the skew distortion. It can be seen that the skew distortion increases from 6.38 to 9.09% when the incident angle increases from 20° to 30° while keeping the MEMS mechanical deflection angle at −5°∼5°. The deflection of the 2D MEMS micromirror has the problem of deflection order of two axes; if the order is different, it will lead to different normal unit vectors and then lead to different point distribution effects of the reflection angle. In this paper, the default MEMS micromirror is deflected horizontally before deflecting vertically, and the order may change in practice, but the analysis method is consistent.

**Figure 7:**
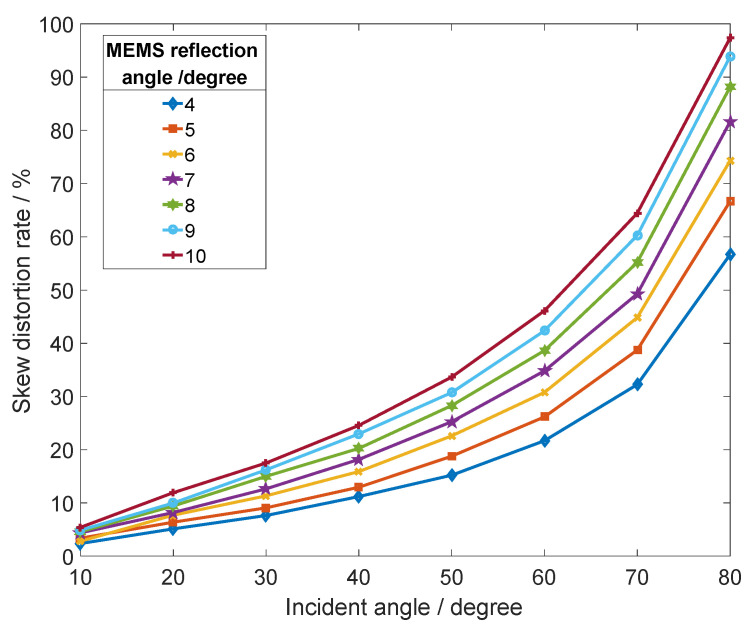
Skew distortion rates at different incidence angles and different deflection angles of MEMS micromirrors.

To study the effect of skew distortion at different MEMS deflection angles and different incident angles, the micromirror deflection angle and light incident angle are changed, respectively, to obtain the skew distortion curve shown in Figure 7. When the deflection angle of the micromirror is constant, the effect of tilt distortion becomes more obvious with the increase of the incident light angle. When the angle of incident light is greater than $50^{{}^{\circ}}$, the skew distortion effect will increase significantly. When the incident light angle is less than 10°, the skew distortion effect does not change significantly with the MEMS micromirror deflection. It can be concluded that the change of the incident angle mainly brings about the skew distortion. When controlling the small amplitude vibration of the MEMS micromirror and the deviation of the angle between the incident light and the normal unit vector of the reflecting surface, the effect of skew distortion on the plane scanning waveform can be effectively reduced.

**Figure 8:**
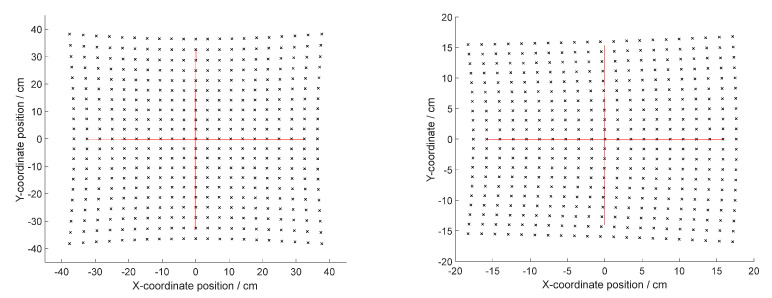
The point cloud distribution of the scanning plane at different MEMS vibration angles and different light incidence angles (left image: pillow distortion effect; right image: skew distortion effect).

From Equation (11) in Section 2.1, it can be seen that the distribution of the point cloud after the 2D MEMS micromirror scanning is different for different deflection angles of the MEMS reflecting surface, different incident angles, and different distances. As shown in Figure 8, at a scanning distance of 1 m, the incident light is incident on the MEMS reflection plane, the deflection angle of the MEMS micromirror is within the variation range of −10°∼10, and the pillow distortion of the point cloud can be obtained as 5%. At the same scanning distance, the incident light is incident on the MEMS reflection plane at an angle of 25°, and the skew distortion of the scanning plane point cloud can be obtained as 8.62%. It can be seen that at a certain scanning distance, the distribution of point cloud of the planar scanning also shows the pillow distortion and skew distortion effects.

## 3. Experimental Demonstration Based on Two-Dimensional MEMS Micromirror Scanning

**Figure 9:**
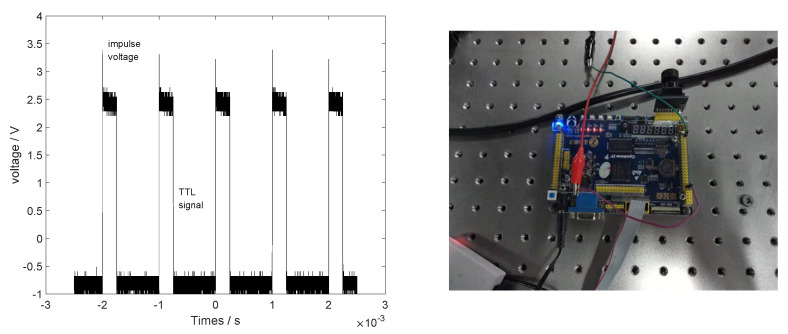
TTL signal generation system (left figure: TTL signal + laser impulse; right figure: TTL circuit).

**Figure 10:**
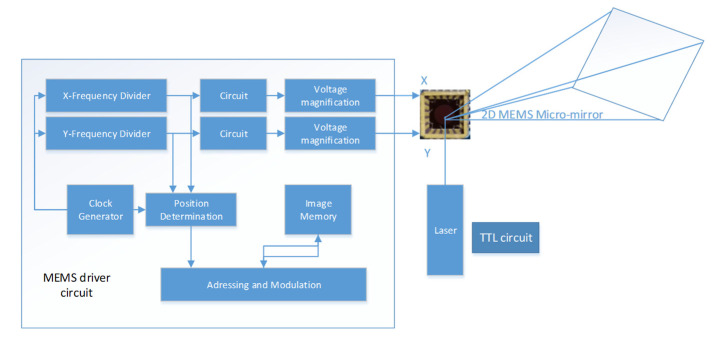
Block diagram of hardware overall design.

To verify the correctness of distortion theory in 2D MEMS planar scanning, a 2D MEMS micromirror scanning platform was built. In this experiment, the micromirror produced by MT company was used; the model is S30859, the diameter is 3.6 mm, and the maximum deflection angle is 6.45° [12]. A 532 nm laser source with a tunable repetition frequency of 1–4 kHz was used. The laser source was controlled by an external TTL signal with adjustable frequency and duty cycle through an external TTL circuit. The laser pulse repetition frequency was set to 1 kHz, and the duty cycle was set to 25%; the TTL signal generation system is shown in Figure 9, from which it can be seen that the TTL circuit controls the pulsed laser. The micromirror was scanned horizontally at 100 Hz and vertically at 20 Hz to obtain a 10×50 scanning dot-matrix waveform with resolution. A projection scanning system was set up for practical verification, consisting of a control and an output control unit. The hardware block diagram is shown in Figure 10.

**Figure 11:**
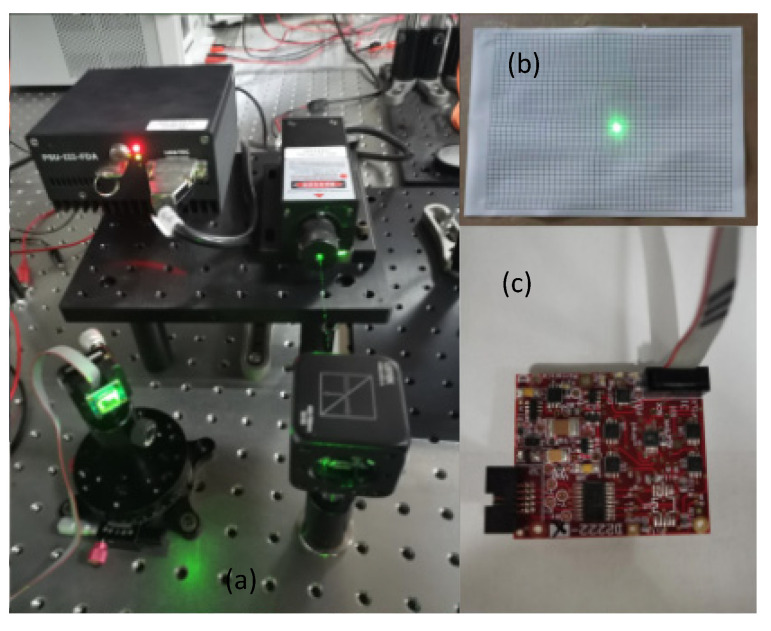
Scanning projection system: (**a**) scanning optical path; (**b**) scanning objective plane; (**c**) MEMS driver.

The MEMS micromirror control circuit uses the MEMS driver board from MTI, composed of three main parts: a quad DAC chip, a low-pass filter, and an amplifier circuit. The quad DAC chip AD5664R can read SPI communication data and then output four voltage signals to drive the MEMS micromirror for two-dimensional deflection. The angle adjuster is used to control the incident angle of 45° with the laser beam splitter. The purpose of the beam splitter is to reduce the output energy source of the laser to protect the MEMS micromirror. At the same time, the output light paths after the laser are perpendicular to each other, and the incident angle can be adjusted to a more accurate position. After the beam is reflected by the stationary MEMS micromirror, the reflected spot is illuminated at the center of the target plate at a target distance of 86 cm. The scanning optical path and MEMS driver module are shown in Figure 11. The system initialization parameters are shown in Table 1.

## 4. Test and Discussion

**Figure 12:**
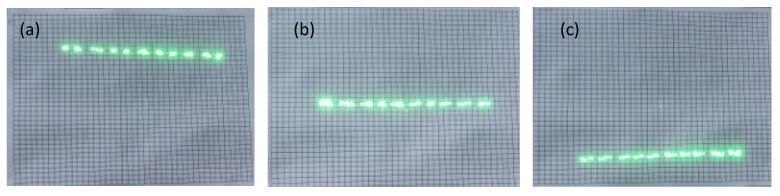
Scanning projection effect: (**a**) distribution of scanning points in the top row; (**b**) distribution of scanning points in the middle row; (**c**) distribution of scanning points in the bottom row.

To highlight the scanning effect, laser scanning was performed in point-to-point mode. According to the scanning point array distribution, the trend of scanning point movement can be seen, thus more intuitively reflecting the scanning distortion. The scanning projection effect is shown in Figure 12. Since the camera shooting speed was slower than the micromirror scanning speed, the top, middle, and bottom three-line scan effect images were selected. Compared to the scanning coordinate plate, the top line scan point distribution shows a downward trend from left to right, the middle line scan point shows a horizontal distribution, and the bottom line scan distribution shows an upward trend from left to right. It shows the skew distortion as described in Section 2.2, and its skew distortion degree is about 8.3%.

In this experiment, we controlled the micromirror deflection angle within ±3° to obtain a better skew distortion effect. Of course, due to the error of applying the laser source spot size and target plate measurement, the measurement result fluctuates ±0.02% with the theoretical analysis value. However, this margin of error is not sufficient to prevent us from making a judgment about the phenomenon and intensity of tilt distortion. The experiments show that our skew distortion effect is in strong agreement with the theoretical analysis.

Our research topic mainly focuses on the optical path design, the micromirror deflection angle, and the incident light angle to build a mathematical model to explore the causes of aberrations. To fully consider the impact of dynamic deformation when building the experimental platform, we selected a 3.6 mm diameter round micromirror lens, compared to the rectangular micromirror surface, diamond-shaped and round micromirror surfaces can greatly reduce the impact of dynamic deformation on the projection imaging effect [13]. In the experiment, the mechanical and physical problems of the micromirror such as mechanical fatigue were kept within a small range; it is known that the long-term use of the micromirror leads to a nonlinear shift of the tilt angle of the micromirror in units of minutes to tens of minutes [14]. To avoid or reduce the occurrence of this phenomenon, our experimental design was implemented in a fast projection display with a scanning projection period of 0.25 s. In this scanning time, the nonlinear shift of the tilt angle of the microscope due to long-term use of the microscope is well avoided. The experimental effect is more prominent and well verifies the effect of the incidence angle on the tilt distortion proposed in our theory.

## 5. Conclusions

The distortion problems of 2D MEMS micromirrors in applications of scanning projection were analyzed; the causes of distortion were elaborated; the types of scanning projection distortion were discussed; and the relationship between the vibration angle of the MEMS micromirror, the angle of light incidence, and the scanning projection distance was constructed. The angle distribution of the incident light and the point cloud distribution of the scanning plane were compared under different conditions. The degree of pillow distortion and skew distortion under different working conditions were demonstrated. Theoretical analysis and experiments show that when the incident angle and the deflection angle of the mirror are small, the influence of tilt distortion and pillow distortion on the projection pattern can be reduced effectively. The existing 2D MEMS scanning micromirror was used to build a scanning projection system, which successfully realized the scanning projection of the preset waveform, highlighted the distortion in the scanning projection, and verified the correctness of the theoretical analysis. It provides theoretical support for the subsequent in-depth study of the application and improvement of scanning projection based on the 2D MEMS micromirror. It also lays the foundation for subsequent research on the correction of scanning distortion.

## Figures and Tables

**Table 1:** Initialization parameter of the scanning projection system.

Initialization Parameters of the System	
Horizontal scanning frequency of MEMS	100 Hz
Vertical scanning frequency of MEMS	20 Hz
Scanning points	10×50
Scanning mode	Point to point
Laser repetition frequency	1 KHz
Laser power	8 mW
Output spot size	1.09 mm
Scanning distance	86 cm
The angle of the incident ray	45°
The deflection angle of the micromirror	±3°
Coefficient of the beam splitter	50:50

## Supplementary Materials

The following are available online at https://www.mdpi.com/article/10.3390/mi12070818/s1.
