# Supplementary material for: Analysis of Distortion Based on 2D MEMS Micromirror Scanning Projection System
Source: Micromachines (Basel). 2021 Jul 13;12(7):818. doi: 10.3390/mi12070818 (PMC8306571; doi:10.3390/mi12070818)

Device Parameters Summary

Device ID : S30859

Actuator Type: A7B1.1

Actuator Mode : Gimbal-less Dual-Axis Quasistatic

Mirror Type and Size : Bonded mirror of 3.6mm diameter

Mirror Coating : Aluminum

Maximum Mech. Angle - X Axis [degrees] : 6.4551

Maximum Mech. Angle - Y Axis [degrees] : 6.4310

Maximum Vdifference - X Axis [V] : 122

Maximum Vdifference - Y Axis [V] : 121

Driver Bias Voltage (Vbias) [V] : 80

Maximum Mech. Angle - Coupled Axes [degrees] : 8.1030

Resonant Frequency - X Axis [Hz] : 300

Resonant Frequency - Y Axis [Hz] : 300

Quality Factor - X Axis : 25

Quality Factor - Y Axis : 27

Recommended LPF Cutoff Frequency (6th Order Bessel) [Hz] : 130

Date and Time Report was Created: 05-Feb-2019 at 11:33:7

Static Response

Voltage vs. Mech. Angle - Individual Axes

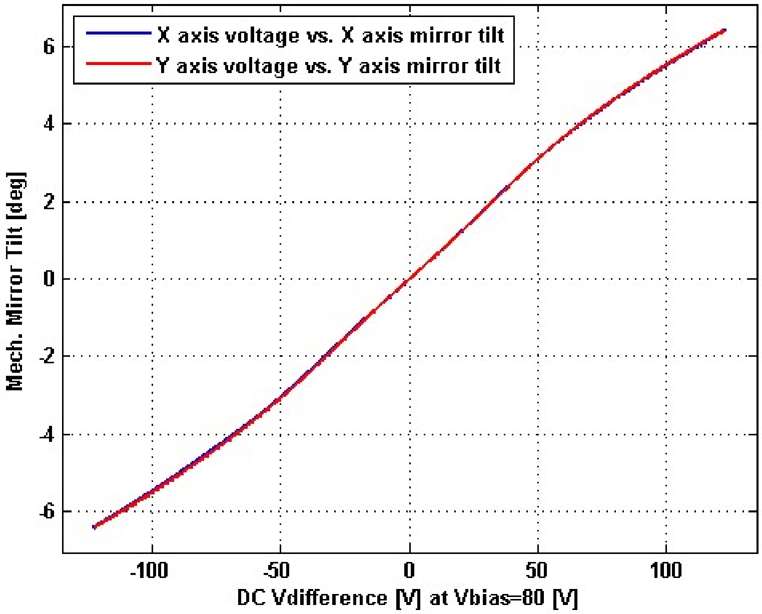

Voltage vs. Mech. Angle - Coupled Axes

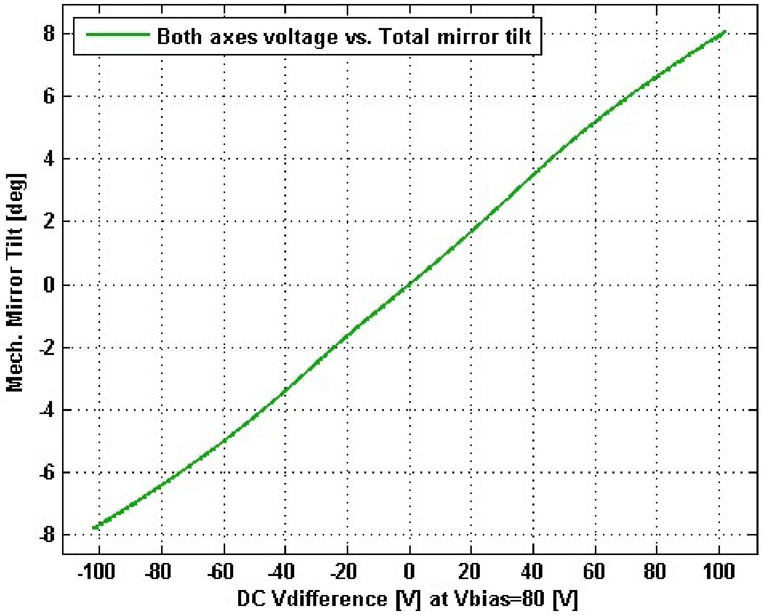

Frequency Response

Small Signal Frequency Response - Magnitude

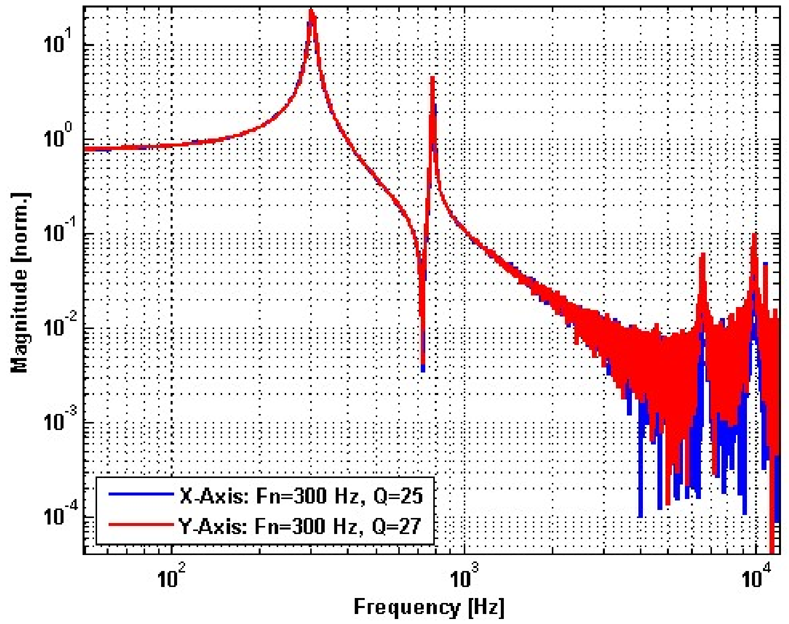

Small Signal Frequency Response - Phase

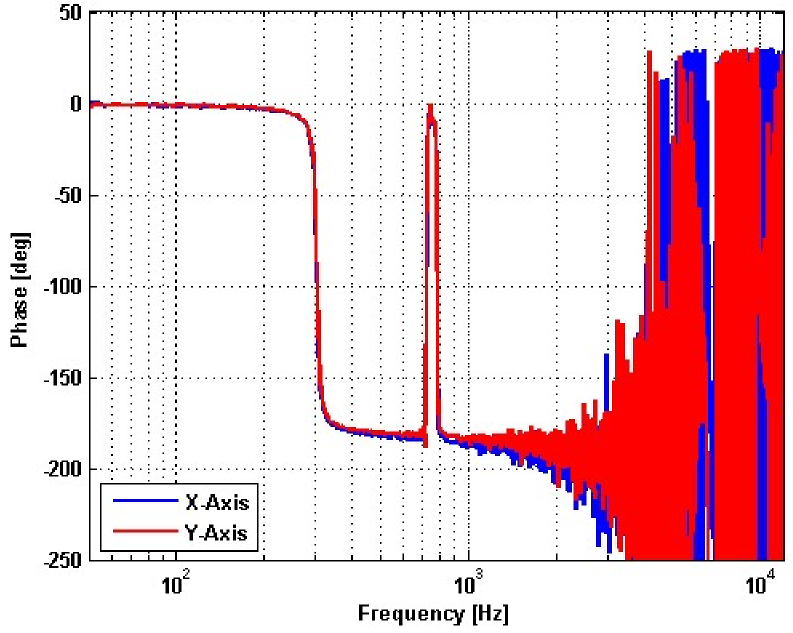

Step Response

X-Axis Step Response

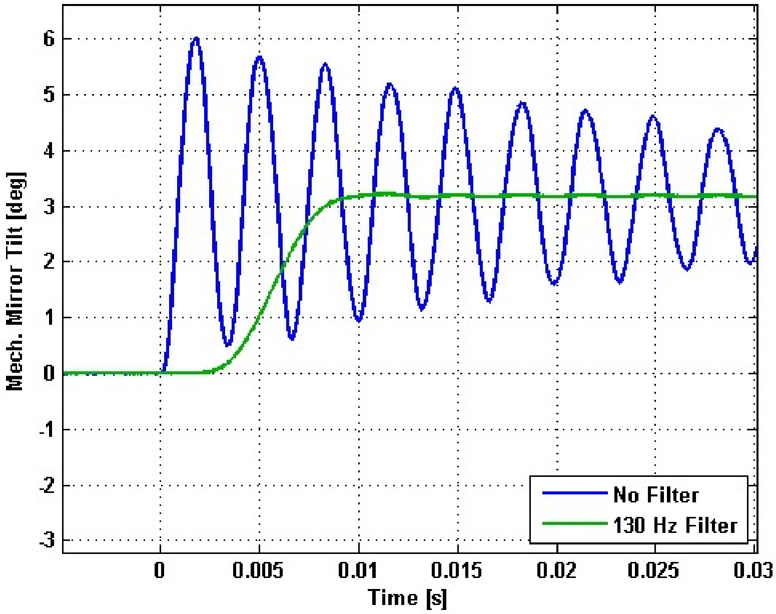

Y-Axis Step Response

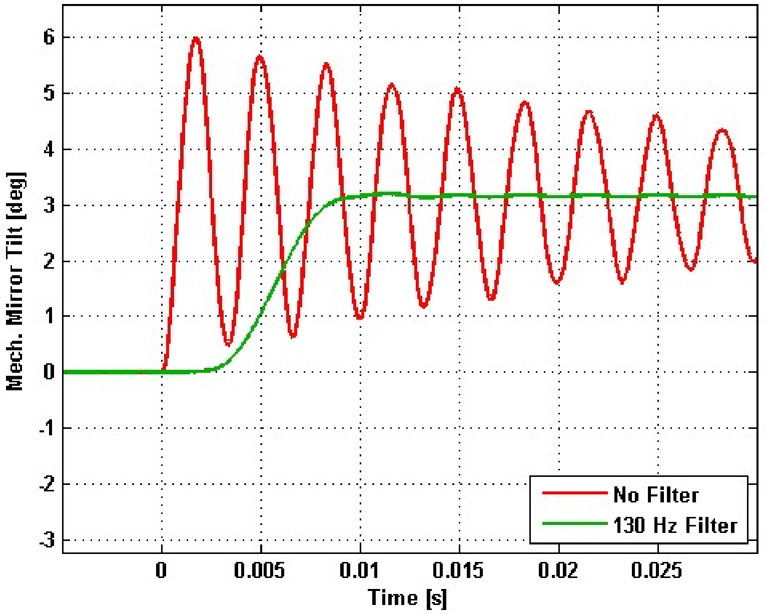

2D Lookup Tables

Voltage vs. X Mech. Angle - Lookup Table

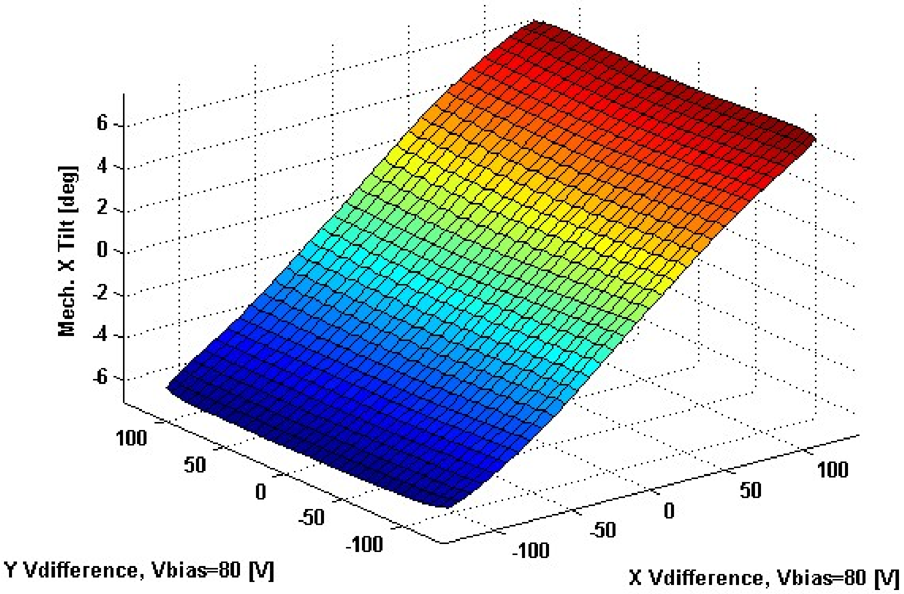

Voltage vs. Y Mech. Angle - Lookup Table

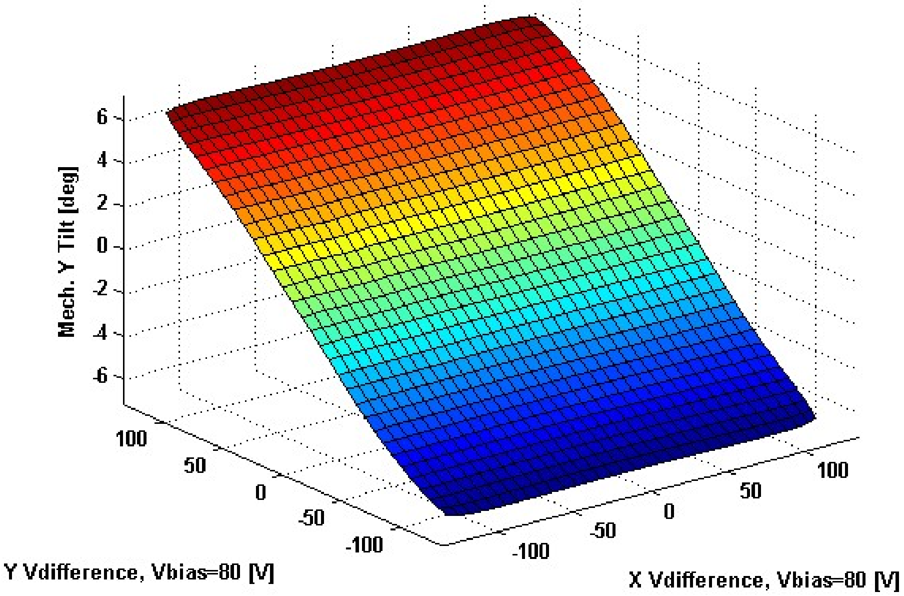

Y vs. X Tilt - Addressable Mech. Angles

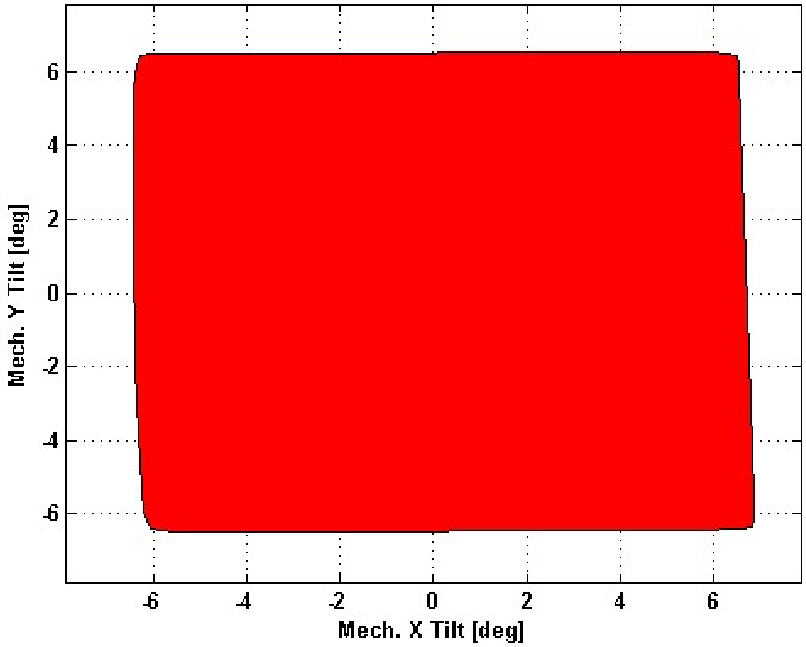

Supplement: Supplementary file 1 [file micromachines-12-00818-s001.zip › micromachines-1260196-supplementary.pdf]
